# SOLAMEN syndrome with cardiovascular damage

**DOI:** 10.1186/s41065-024-00314-2

**Published:** 2024-07-30

**Authors:** Xiong Zhao, Xiaojie Yue, Shifan Yuan, Yefeng Dai, Hao Gu

**Affiliations:** 1 Department of Burn and Plastic Surgery, The Children’s Hospital, National Clinical Research Center, Zhejiang University School of Medicine, Zhejiang Hangzhou, PR China; 2 Shanghai ninth People’s Hospital, Shanghai Jiao Tong University of medicine, Shanghai, PR China

**Keywords:** SOLAMEN syndrome, Arteriovenous malformation, PTEN mutation, Segmental overgrowth, Lipomatosis

## Abstract

SOLAMEN syndrome is a rare, recently recognized congenital syndrome that is characterized by progressive and hypertrophic diseases involving multiple systems, including segmental overgrowth, lipomatosis, arteriovenous malformation (AVM) and epidermal nevus. According to literatures, SOLAMEN syndrome is caused by heterozygous PTEN mutation. Phenotypic overlap complicates the clinical identification of diseases associated with PTEN heterozygous mutations, making the diagnosis of SOLAMEN more challenging. In addition, SOLAMEN often presents with segmental tissue overgrowth and vascular malformations, increasing the possibility of misdiagnosis as klipple-trenaunay syndrome or Parks-Weber syndrome. Here, we present a case of a child presenting with macrocephaly, patchy lymphatic malformation on the right chest, marked subcutaneous varicosities and capillaries involving the whole body, overgrowth of the left lower limb, a liner epidermal nevus on the middle of the right lower limb, and a large AVM on the right cranial thoracic entrance. Based on the typical phenotypes, the child was diagnosed as SOLAMEN syndrome. detailed clinical, imaging and genetic diagnoses of SOLAMEN syndrome was rendered. Next-generation sequencing (NGS) data revealed that except for a germline PTEN mutation, a PDGFRB variant was also identified. A subsequent echocardiographic examination detected potential cardiac defects. We suggested that given the progressive nature of AVM and the potential severity of cardiac damage, regular echocardiographic evaluation, imaging follow-up and appropriate interventional therapy for AVM are recommended.

## Background

SOLAMEN syndrome is a rare, recently recognized congenital disease that is associated with a heterozygous pathogenic mutation in PTEN and was firstly identified by Caux F in 2007 [1, 2]. It is characterized by progressive and hypertrophic disorders involving multiple systems, including segmental overgrowth, lipomatosis, arteriovenous malformation (AVM) and epidermal nevus [2, 3]. However, due to its rarity, SOLAMEN patients are often misdiagnosed as klipple-trenaunay syndrome (KTS) or Parks-Weber syndrome (PWS), based on the clinical phenotypes of the segmental tissue overgrowth and vascular malformations. Besides, of phenotypes of SOLAMEN syndrome strongly overlap with those of PTEN hamartoma syndromes. Heterozygous germline tumor-suppressor PTEN mutations are associated with Cowden syndrome (MIM 158,350), and Bannayan-Riley-Ruvalcaba syndrome (MIM 153,480) [1, 4–7]. Neoplastic lesions involving multiple systems are the common phenotypes of PTEN hamartoma syndromes. Therefore, phenotypic overlap complicates the clinical identification of diseases associated with PTEN heterozygous mutations, making the diagnosis of SOLAMEN more challenging. The clinical phenotypes of SOLAMEN syndrome mimicked Proteus syndrome, but is distinct from either Cowden syndrome or Bannayan-Riley-Ruvalcaba syndrome. A comprehensive clinical examination can provide more information for differential diagnosis.

Here, we report a patient presented with macrocephaly, diffuse abdominal lipomatosis, patchy lymphatic malformation on the right chest, marked subcutaneous varicosities and capillaries involving the whole body, overgrowth of the left lower limb, foot and hand hypertrophic malformations, a liner epidermal nevus on the middle of the right lower limb, subclavian AVM and impaired left-cardiac function. We identified de novo pathogenic mosaic variants in PDGFRB (c.2T > G) and PTEN (c.962delC) and raised the diagnosis of SOLAMEN syndrome.

## Case report

**Fig. 1:**
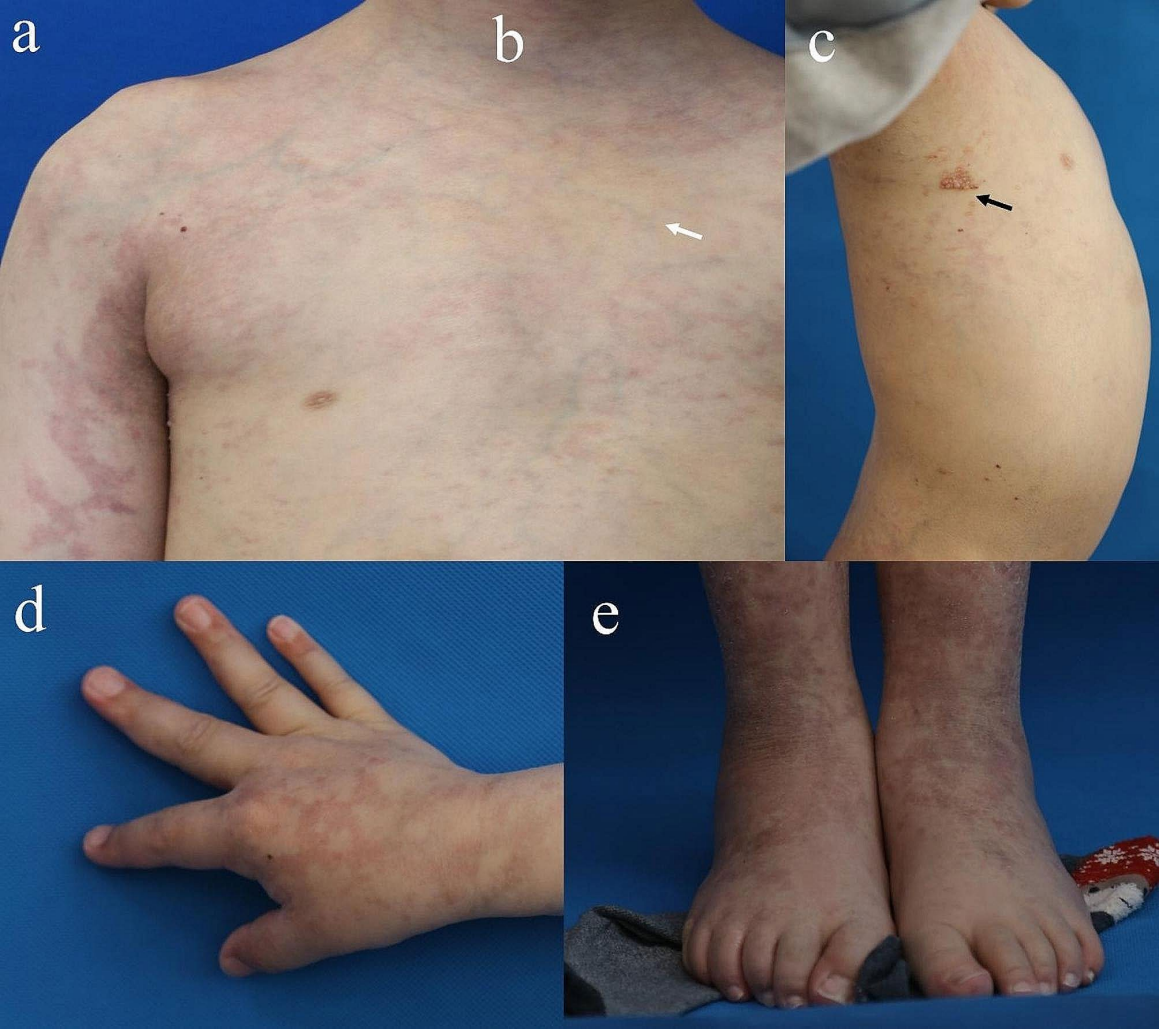
Clinical phenotypes of the patient. (**a**) An 11-year-old male patient presented with marked subcutaneous varicosities and capillaries involving the whole body and severe abdominal distension. (**b**) Overgrowth of the left lower limb and a liner epidermal nevus on the middle of the right lower limb (white arrow). (**c**) Skin images showed a large patchy lymphatic malformation (LM) on the right chest (black arrow). (**d-e**) Hypertrophy of right hand and foot

**Fig. 2:**
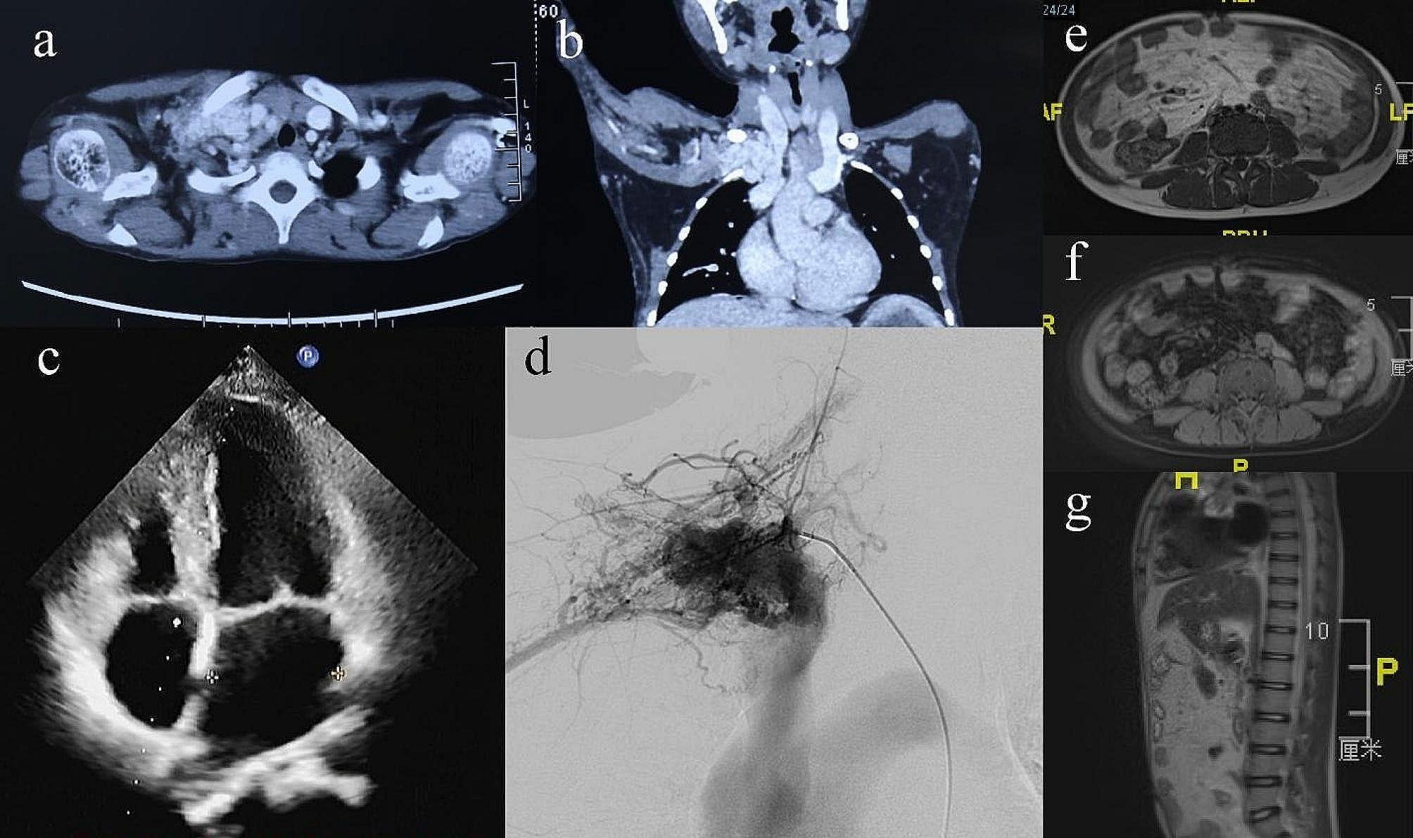
Computed tomography (CT), echocardiography, digital substraction angiography (DSA) and magnetic resonance imaging (MRI) images of the patient. (**a-b**) CT scan (coronal and axial images) showed a large AVM on the right cranial thoracic entrance and enlargement of the cardiac shadow. (**c**) echocardiography demonstrated an enlarged left ventricle and left atrium. (**d**) DSA showed a high-flow AVM, fed by the right subclavian artery and its branches. (**e-g**) MRI (T1WI axial, T1WI axial with fat-suppression and T1WI sagittal images) showed diffuse fatty infiltration of the abdomen

An 11-year-old male patient with an unremarkable family history was referred to our clinic for a mass on the right chest with persistent pulsation. Further physical examination showed macrocephaly with an occipitofrontal circumference (OFC) measurement of 56.7 cm, severe abdominal distension, a large patchy lymphatic malformation (LM) on the right chest, marked subcutaneous varicosities and capillaries involving the whole body, overgrowth of the left lower limb, a liner epidermal nevus on the middle of the right lower limb, and right foot and hand hypertrophic malformations (Fig. 1a-e). Oral examination did not revealed mucosal papules. An enhanced computed tomography (CT) scan revealed a large AVM on the right cranial thoracic entrance and enlargement of the cardiac shadow (Fig. 2a-b). Echocardiography demonstrated an enlarged left ventricle and left atrium (Fig. 2c). A transfemoral angiogram showed a right subclavian AVM with feeder vessels of the right subclavian artery and its branches. Small quantities of absolute ethanol were injected through the feeder vessels (Fig. 2d). MR imaging showed diffuse fatty infiltration of the abdomen (Fig. 2e-g).

Following patient consent, we performed minimally invasive needle biopsy punch (D = 2 mm, all layer biopsy) of both the LM and epidermal nevus lesion to further clarify the molecular information. Targeted next-generation sequencing (NGS) was performed on tissue samples and peripheral blood samples across a panel of 40 vascular anomaly-specific genes according to the ISSVA classification [8] with a median target fold coverage of > 10,000×. Molecular analysis showed a germline PTEN pathogenic variant ((NM 000314.8, c.962delC, p.Thr321fs) that had previously been identified in SOLAMEN [2] and a germline mutation of the PDGFRB gene (NM 001355016.2, c.2T > G). Sequencing of DNA from the LM lesion and epidermal nevus lesion of the patient confirmed these two germline variants (Table 1). Neither the PTEN nor PDGFRB variant were identified in the proband’s parents and were considered de novo mutations. According to the diagnostic criteria for SOLAMEN syndrome [2], we diagnosed the child with SOLAMEN syndrome based on the clinical phenotypes, including subclavian AVM, diffuse infiltrating abdominal lipomatosis, right chest LM, segmental overgrowth of lower limb and linear epidermal nevus, and the genetic study that revealed a germline PTEN pathogenic variant.

**Table 1:** Detailed genetic information of the patient

Sample	gene	chr:posi	ref > alt	geno	depth(RD + AD)	mutation frequency	Impact	annotation	hgvs_c	hgvs_p
Blood	PDGFRB	5:149516609	A > C	0*1	1614 + 1544	0.4875	HIGH	start_lost	c.2T > G	p.Met1?
	PTEN	10:89720810	AC > A	1*0	3591 + 4059	0.5268	HIGH	frameshift_variant	c.962delC	p.Thr321fs
LM lesion	PDGFRB	5:149516609	A > C	0*1	1331 + 1167	0.4657	HIGH	start_lost	c.2T > G	p.Met1?
	PTEN	10:89720810	AC > A	1*0	1030 + 2861	0.7295	HIGH	frameshift_variant	c.962delC	p.Thr321fs
epidermal nevus lesion	PDGFRB	5:149516609	A > C	0*1	1067 + 1004	0.4841	HIGH	start_lost	c.2T > G	p.Met1?
	PTEN	10:89720810	AC > A	1*0	1606 + 1659	0.5067	HIGH	frameshift_variant	c.962delC	p.Thr321fs

## Discussion

SOLAMEN syndrome (Segmental Overgrowth, Lipomatosis, Arteriovenous Malformation and Epidermal Nevus) was first proposed in 2007 to describe a PTEN-related syndrome distinct from Proteus syndrome [2]. SOLAMEN syndrome was previously considered as the segmental exacerbation of Cowden syndrome, and was reported in patients with positive family history of Cowden syndrome. Here, we report a rare SOLAMEN syndrome without family history of Cowden disease showing an extensive subcutaneous varicosities and capillaries involving the whole body, lymphatic malformation, subclavian AVM, asymmetric hypertrophy of the left leg, localized epidermal nevus, and diffuse abdominal lipomatosis diagnosed by a multidisciplinary team of expert radiologists and geneticists. Sequence analysis from DNA extracted from both the LM lesion, the epidermal nevus lesion and peripheral blood shows a profile of a germline PTEN pathogenic variant (c.962delC, p.Thr321fs) and a germline PDGFRB (c.2T > G) variant.

AVM, a fast-flow vascular anomaly that consist of abnormal artery-vein bypasses without intervening capillary network, and they comprise 4.7% of all vascular anomalies [9, 10]. The fast-flow supplying arteries and draining veins of AVM is prone to steal blood, which results in pain, hemorrhage, ulcers, deformities, chronic volume overload of the heart, ventricular dilatation, and heart failure [9–11]. AVMs are progressive throughout life, and most of them begin during childhood and adolescence [12]. Traditional therapies, such as embolization and excision provide only transient benefits. Thoracic AVM is particularly rare, leading to impairment of heart function in an early stage [13]. The PDGFRB gene plays an important role in cardiovascular development [14, 15]. Mutant Pdgfb mice can display structural cardiovascular anomalies, including enlarged and deformed hearts [16].

Based on the limited number of patients described thus far, it remains to be elucidated whether the impaired left-cardiac function is an AVM-associated or variant-specific feature of PDGFB that warrants routine additional evaluation. Given the progressive nature of AVM and the potential severity of cardiac damage, regular echocardiographic evaluation, imaging follow-up and appropriate interventional therapy for AVM are recommended.

The overlapping clinical phenotypes of several asymmetric overgrowth syndromes such as Proteus syndrome have caused many patients to be misdiagnosed. Proteus syndrome is a rare complex disorder associated with ATK1 and PTEN mutations, and the overgrowth of Proteus syndrome is typically distorting, disproportionate and asymmetric [17]. Vascular malformations are the most distinctive feature in SOLAMEN syndrome. Vascular malformations are thought to be caused by abnormal signaling processes during embryogenesis, leading to the persisting vascular cells with certain degree of differentiation [18]. Vascular malformations, consisting of abnormally formed blood vessels that are lined by endothelial cells, are separated into two major categories: fast-flow malformations and low-flow malformations [19, 20]. AVMs and arteriovenous fistula(AVF) are the major components of fast-flow malformations. As for low-flow malformations, apart from the capillary malformations(CMs), venous malformations(VMs), LMs, and lymphatico-venous malformations (LVMs) become the most part. VM, LM, and LVM were vascular anomalies which were congenital, often unnoticed at birth, progressing proportionally with individuals, and never self-regress. SOLAMEN patients are often misdiagnosed as vascular malformation syndromes including KTS or PWS, based on the clinical phenotypes of the segmental tissue overgrowth and vascular malformations.

KTS was firstly described by Klippel M and Trenaunay P in 1900 as a new syndrome associated with vascular malformations and limb hypertrophy [21]. It is now recognized that KTS is one of segmental overgrowth phenotypes vascular malformations due to PIK3CA mutation, as called PIK3CA-related overgrowth spectrum (PROS) [22, 23]. The diagnosis of KTS is mainly based on phenotypes including CMs, lymphatic and venous malformations, and tissue hypertrophy on the affected extremity. CLOVES syndrome (Congenital Lipomatous Overgrowth, Vascular malformations, and Epidermal nevi ) is an overgrowth syndrome caused by mosaic activating mutation in gene PIK3CA, which is also designated as PROS [24]. The typical vascular anomalies of CLOVES syndrome are usually lymphovascular malformations. Another overgrowth syndrome, PWS is characterized by extensive CMs, segmental overgrowth extremity and AVMs/AVFs [25]. Individuals with PWS usually present with progressive overgrowth of affected extremity through life. The diagnosis of KTS, PWS and SOLAMEN syndrome, mainly based on physical findings e can be challenging as some phenotypes are difficult to recognize in young children, leading to be overlooked in the early stage. These conditions aware us that the differentiation among KTS, PWS and SOLAMEN syndrome is particularly important. Causative gene mutations genes and beginning of elucidation of the pathophysiological pathways of most vascular malformations has been unraveled [26]. KTS is resulted from abnormal PI3K-AKT‐mTOR pathway activation, [22, 27] while PWS frequently harbors somatic activating mutations in the RAS-RAF-MAPK pathway [28]. We propose that apart from careful physical examination and medical history, genetic analysis is recommended when diagnosis is challenging in an early stage, provide more information to comprehensive understanding of KTS.

mTOR is a serine/threonine kinase regulated by PI3K. Sirolimus, an allosteric mTOR inhibitor can thereby prevent downstream protein synthesis and subsequent cell proliferation and angiogenesis in which the mTOR growth control pathway is affected [29, 30]. Since PIK3CA variants are found in the majority of KTS, sirolimus is predicted to be efficient agents for medical treatment of KTSs with lymphatic component by directly inhibiting mTOR. The use of sirolimus in PTEN hamartoma tumor syndrome have also been reported [31]. Sirolimus is expected to neutralize the impact of PTEN loss, thereby improving the symptoms [32]. PIK3CA inhibitors such as BYL719 are also expected to be effective in particular on vascular malformations of SOLAMEN [33].

## Conclusion

Here, we present a child patient presented with macrocephaly, diffuse abdominal lipomatosis, patchy lymphatic malformation on the right chest, marked subcutaneous varicosities and capillaries involving the whole body, overgrowth of the left lower limb, hypertrophy of right hand and foot, a liner epidermal nevus on the middle of the right lower limb, subclavian AVM and impaired left-cardiac function, and diagnosed as with SOLAMEN syndrome.

Detailed clinical, imaging and genetic diagnoses of SOLAMEN syndrome was rendered. NGS data revealed that in addition to a germline PTEN (c.962delC) mutation, a de novo pathogenic mosaic PDGFRB (c.2T > G) variant was also identified. Given the progressive nature of AVM and the potential severity of cardiac damage, regular echocardiographic evaluation, imaging follow-up and appropriate interventional therapy for AVM are recommended. Rapamycin targeted therapy may benefit SOLAMEN patients with cardiac involvement.

## Data Availability

All supporting data of this article are included in the submitted manuscript.

## Abbreviations

AVM: Arteriovenous malformation
KTS: Klipple-trenaunay syndrome
PWS: Parks-Weber syndrome
LM: Lymphatic malformation
CT: Computed tomography
DSA: Digital substraction angiography
MRI: Magnetic resonance imaging
NGS: Next-generation sequencing
AVF: Arteriovenous fistula
CMs: Capillary malformations
VMs: Venous malformations
LVMs: Lymphatico-venous malformations
PROS: PIK3CA-related overgrowth spectrum
